# Triterpene Content in Flesh and Peel of Apples Grown on Different Rootstocks

**DOI:** 10.3390/plants11091247

**Published:** 2022-05-05

**Authors:** Aurita Butkevičiūtė, Valdimaras Janulis, Darius Kviklys

**Affiliations:** 1 Department of Pharmacognosy, Lithuanian University of Health Sciences, Sukileliu Ave. 13, 50162 Kaunas, Lithuania; valdimaras.janulis@lsmuni.lt; 2 Institute of Horticulture, Lithuanian Research Centre for Agriculture and Forestry, Kauno St. 30, 54333 Babtai, Lithuania; darius.kviklys@lammc.lt; 3 Department of Horticulture, Norwegian Institute of Bioeconomy Research—NIBIO Ullensvang, Ullensvangvegen 1005, 5781 Lofthus, Norway

**Keywords:** apple, food quality, Malus, triterpene

## Abstract

Advancements in rootstock breeding and selection have revolutionized the manner in which apples are grown throughout the world. Fruit tree breeding has typically focused on key horticultural characteristics. Even though agents with health benefits have been investigated more frequently during the recent years, information about the effect of different cultivation factors, such as the rootstock, on triterpene concentration is still lacking. The present study aimed to evaluate triterpene profiles and the quantitative composition of different parts of apple fruit that was grown on 17 various origin and vigor rootstocks. HPLC analyses of triterpenes in apple samples were performed. The highest total content of triterpenes (7.72 ± 0.39 mg/g) was found in peel samples of apples grown on the dwarf rootstock 62-396-B10^®^. Depending on the rootstock, apple peel samples accumulated 3.52 to 4.74 times more triterpene compounds than apple flesh samples. Ursolic acid was the predominant triterpene compound in apple peel and flesh samples. The highest content of ursolic acid (5.84 ± 0.29 mg/g) was found in peel samples of apples grown on the dwarf rootstock 62-396-B10^®^. Meanwhile, the lowest amount of ursolic acid (3.25 ± 0.16 mg/g) was found in apple peel samples grown on the dwarf rootstock Cepiland-Pajam^®^2. A proper match of a cultivar and a rootstock can program a fruit tree to grow larger amounts of higher quality, antioxidant-rich, and high-nutrition-value fruit.

## 1. Introduction

The sector of fruit production is a relevant part of agriculture and in Europe makes up 6.70% of the total agricultural production. Apple orchards compose a little over one-third (36.60%) of the total area of fruit plantations in Europe. The largest apple producers in Europe are Poland (25.00%), Italy (19.20%), and France 15.50% [1]. Consequently, apples remain one of the most widely grown and consumed fruits in the world, and their processed products are widely used in the production of food supplements, food industry products, and beverages [2,3,4,5]. 

Apples have a varied and well-balanced complex of biologically active compounds such as vitamins, minerals, trace elements, fibers, and organic acids [6]. Furthermore, apples are considered an important source of triterpene and phenolic compounds [3]. Research into other important bioactive compounds including triterpenes in apples is performed to a much lesser extent. Pentacyclic triterpenes are notably present in plant surfaces such as stem bark, leaves, and fruit waxes, where they protect the plant from biotic and abiotic stress factors. The qualitative and quantitative composition of triterpene compounds vary in the peel and flesh of apples. The cuticular wax of apple peel represents an important food source of triterpenes [7]. Previous studies have described the antioxidant [8], antimicrobial [9,10], antidiabetic [11,12], and anticancer [13,14] potential of triterpenes. Other studies have also reported their anti-inflammatory, analgesic, hepatoprotective, cardiotonic, and sedative activity [15,16,17]. Triterpenes are a group of biologically active compounds with a wide range of biological activities, and their preparations can be used for the prevention of chronic diseases [11].

Agrotechnology of fruit tree cultivation, climatic conditions, soil composition, UV radiation, and the location of the fruit in the fruit tree crown may influence changes in the content of biological active compounds in apple fruit [18,19,20]. Apple cultivar is one of the main factors affecting the composition of biologically active compounds in apples and the quality of the fruit [21]. Due to the lack of success of many governmental programs for increasing consumption of fruit and vegetables, it has been suggested that increased concentration of biologically active compounds and nutrients by breeding programs could lead to improved health [22]. However, to elevate health-enhancing substances within plants, a better understanding of the effects of cultivation and other environment factors is needed [23]. Advancements in rootstock breeding and selection have revolutionized the manner in which apples are grown throughout the world [24]. Rootstocks significantly affect the growth properties of fruit trees, the onset of fruiting, the yield, and resistance to frost and drought, and establish the organoleptic properties and mineral composition of the fruit [25,26,27,28]. Rootstocks have also been found to affect the qualitative and quantitative content of phenolic compounds in apples [29,30,31]. Generally, rootstocks can significantly influence a great number of important growing properties of fruit trees, and in some fruit species, their influence accounts for about 50.00% of the economic results [32]. 

Fruit tree breeding has typically focused on key horticultural characteristics including yield, pest and disease resistance, and fruit qualities such as attractiveness and taste. However, consumers are increasingly purchasing fruits for their perceived health-promoting properties. For example, purchases may be influenced by the composition of biologically active compounds, such as phenolic and triterpene compounds, in the fruit. Even though compounds with health benefits have been investigated more frequently during the recent years, information about the effect of different cultivation factors, such as the rootstock, on triterpene concentration is still lacking. Rootstock is an important feature of apple trees in modern orchards. A properly selected cultivar and rootstock can program the fruit tree to produce more high-quality and high-nutrition-value fruits [33]. In recent years, a relatively high number of new apple rootstocks have been delivered by breeding centers around the world. These rootstocks outperform traditionally used rootstocks in productivity and resistance to various abiotic and biotic stresses. Until now, research has been focused on biologically active compounds such as phenolic compounds or endogenous hormones, mineral nutrients, carbohydrate levels, and scion growth of apples grafted onto different rootstocks [34]. We did not find any research data about other important bioactive compounds including triterpenes in apples grown on different rootstocks. It was thus urgent to assess the variation in the qualitative and quantitative composition of individual triterpene compounds in apple flesh and peel samples and identify apple rootstocks that determine the highest triterpene content in apples and the strongest antioxidant activity in vitro. The results of the research are important and allow for evaluation of the effect of apple rootstocks on the qualitative and quantitative composition of individual triterpene compounds. The obtained data are important from a practical point of view, as they allow for selecting optimal combinations of apple cultivars and rootstocks and creating modern garden structures in order to provide consumers with larger amounts of apples, food products, and food supplements with a known composition of biologically active compounds.

The present study aimed to evaluate triterpene profiles and the quantitative composition of different parts of apple fruit that was grown on 17 various origin and vigor rootstocks. 

## 2. Results

### 2.1. Influence of Rootstocks on the Triterpene Content in Apple Peel and Flesh Samples

In horticulture, the use of rootstock is an effective way to control tree growth and yield, to improve resource use efficiency, and to confer scions with desired traits of resilience against a variety of internal and environmental stressors [35]. Even though phenolic compounds with health benefits have been investigated more frequently during the recent years, data about the effect of different cultivation factors, such as the rootstock, on triterpene content are still scarce. The qualitative and quantitative composition of biologically active compounds vary depending on the parts of the apple fruit, i.e., the flesh and the peel [36]. The highest content of lipophilic biologically active compounds is stored in the waxy layer of the cuticle of apple peel [7,37]. In fruit, the bulk of the triterpenes are located in the waxy cuticle layer on the surface of the peel [38]. The review of the scientific literature did not yield any studies describing the influence of rootstocks on the qualitative and quantitative composition of the triterpene compounds in different parts of the fruit, i.e., the peel and flesh. It was thus urgent to determine how rootstocks of different origins and growth affect the qualitative and quantitative composition of pentacyclic triterpenes (ursolic, oleanolic, betulinic, and corosolic acids) in apple samples and identify the most promising rootstocks that ensure the maximum amounts of biologically active compounds.

**Figure 1:**
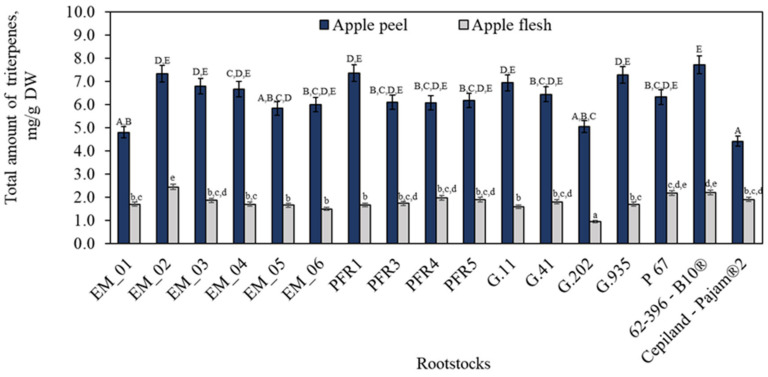
Influence of rootstocks on the quantitative composition of triterpene compounds. The means followed by different uppercase and lowercase letters are significantly different at *p* < 0.05.

Our study was performed on the cultivar ‘Galaval’, which is one of the color mutations of the ‘Gala’ cultivar. The cultivar ‘Gala’ and its clones are widely planted and are some of the world’s top apple cultivars [39,40]. We found that depending on the rootstocks, the total amount of triterpene compounds in the ‘Galaval’ apple peel and flesh samples ranged from 4.41 ± 0.22 mg/g to 7.72 ± 0.39 mg/g and from 0.93 ± 0.05 mg/g to 2.43 ± 0.15 mg/g, respectively (Figure 1). The maximum total content of triterpene compounds (7.72 ± 0.39 mg/g) in apple peel samples was determined in fruit grown on the dwarf 62-396-B10^®^ rootstock. The maximum total content of triterpene compounds (2.43 ± 0.15 mg/g) in apple flesh samples was found in fruit grown on the dwarf EM_02 rootstock. We found that the content of triterpene compounds accumulated in apple peel samples was 3.52–4.74 times higher than that accumulated in apple flesh samples. We found that depending on the rootstock, the content of triterpene compounds in apple peel samples moderately strongly and positively (r = 0.468, *p* <0.001) correlated with the content of these compounds in apple flesh samples. The results of our study confirm the studies which found that the peels of fruits were rich in triterpene compounds and the flesh of fruits contained some amount of triterpene compounds, but their concentrations were much lower than those in peels [41]. According to the data found in the scientific literature, the content of triterpene compounds in apple fruit is 0.28–0.34% of peel dry weight and 18.00–19.50% of peel wax extract [38].

**Figure 2:**
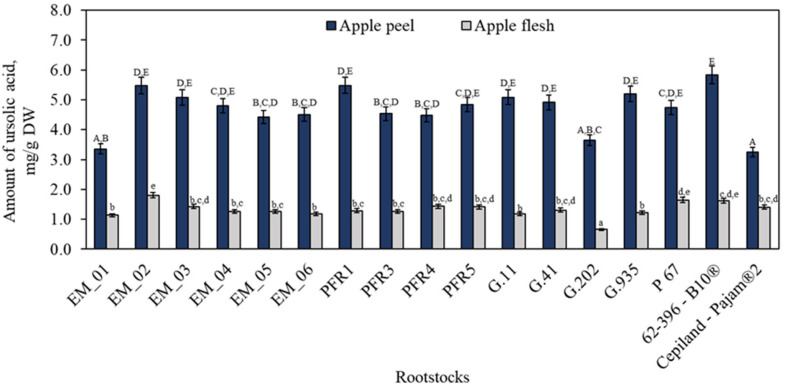
Influence of rootstocks on the quantitative composition of ursolic acid. The means followed by different uppercase and lowercase letters are significantly different at *p* < 0.05.

Apple peel functions as a protective barrier, and thus preserving the integrity and appearance of the peel is critical for market success. In apples, peel epidermal cells and the associated epicuticular wax are rich sources of secondary metabolites [42]. The following triterpene compounds were identified and quantified in the studied apple peel and flesh samples: ursolic, oleanolic, corosolic, and betulinic acids. Depending on the rootstock, the ursolic acid content in the apple peel samples ranged from 3.25 ± 0.16 mg/g to 5.84 ± 0.29 mg/g (Figure 2). The highest content of ursolic acid (5.84 ± 0.29 mg/g) was found in peel samples of apples grown on the dwarf rootstock 62-396-B10^®^. Meanwhile, the lowest amount of ursolic acid (3.25 ± 0.16 mg/g) was found in apple peel samples grown on the dwarf rootstock Cepiland-Pajam^®^2, which did not differ statistically significantly from the amounts found in peel samples of apples grown on the semi-dwarf rootstocks EM_01 or G.202. We found that the ursolic acid content in the apple flesh samples ranged from 0.65 ± 0.03 mg/g to 1.80 ± 0.09 mg/g and was 3.24–4.95 times lower than that found in the apple peel samples (Figure 2). The highest content of ursolic acid (1.80 ± 0.09 mg/g) in apple flesh samples was found in fruit grown on the dwarf rootstock EM_2, which did not differ statistically significantly from the amounts found in apple flesh samples in fruit grown on dwarf rootstocks P 67 or 62-396-B10^®^. The amount of ursolic acid in apples depends on the genotype of the cultivar. In our study, apple peel samples of the ‘Galaval’ cultivar were found to contain higher levels of ursolic acid compared to 0.14 mg/g found in apple peel samples of the ‘Brookfield Gala’ cultivar [43]. The amount of ursolic acid in 94 different apple cultivars grown in New Zealand ranged from 0.45 mg/g to 3.52 mg/g, reported by Andre et al. [7]. The apple samples of the ‘Aroma’ cultivar grown on the semi-dwarf rootstock MM.106 had higher levels of ursolic acid than the samples of apples grown on the dwarf rootstock M.9, according to the results of a study reported by Lv et al. [23]. We did not find any statistically significant differences in the amounts of ursolic acid between different rootstock growth groups or rootstocks of different origins.

**Figure 3:**
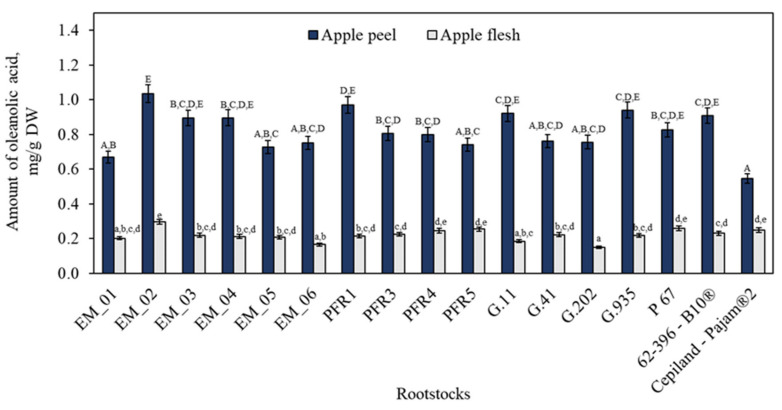
Influence of rootstocks on the quantitative composition of oleanolic acid. The means followed by different uppercase and lowercase letters are significantly different at *p* < 0.05.

Ursolic acid is always accompanied by a minor amount (around one-fifth) of the isomeric oleanolic acid in apple peels [37]. Our study showed that the amount of oleanolic acid in the apple peel samples ranged from 0.55 ± 0.03 mg/g to 1.03 ± 0.05 mg/g, depending on the rootstock (Figure 3). The highest content of oleanolic acid (1.03 ± 0.05 mg/g) in apple peel samples was found in fruit grown on the dwarf rootstock EM_02, while the lowest content (0.55 ± 0.03 mg/g) was found in peel samples of apples grown on the dwarf rootstock Cepiland-Pajam^®^2. In apple flesh samples (as in apple peel samples), the highest content of oleanolic acid (0.30 ± 0.01 mg/g) was found in fruit grown on the dwarf rootstock EM_02, which did not differ statistically significantly from the amount of oleanolic acid detected in apple flesh samples in fruit grown on dwarf rootstocks G.41 or 62-396-B10^®^ (Figure 3). Apple flesh samples in fruit grown on different rootstocks had 3.56 times lower oleanolic acid content compared to apple peel samples. The amount of oleanolic acid as well as that of ursolic acid depends on the genotype of the cultivar. Klein et al. found that the content of oleanolic acid (9.50 mg/g) in the waxy layer of the apple peel of the ‘Royal Gala’ cultivar was higher than that found in the waxy layer of the peel in fruit samples of ‘Granny Smith’, ‘Pink Lady’, or ‘Red Delicious’ cultivars [44]. The amount of oleanolic acid in apple peel samples varied from 0.05 mg/g to 0.84 mg/g, determined by Andre et al. [7]. According to the author, fruits which are characterized by a thicker opaque and waxy peel tended to have higher triterpene acid contents [7].

**Figure 4:**
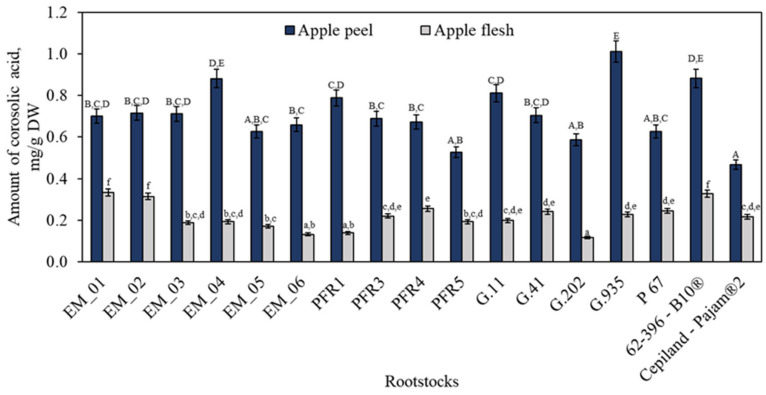
Influence of rootstocks on the quantitative composition of corosolic acid. The means followed by different uppercase and lowercase letters are significantly different at *p* < 0.05.

Depending on the rootstock, the amount of corosolic acid detected in apple peel and apple flesh samples ranged from 0.47 ± 0.02 mg/g to 1.03 ± 0.04 mg/g and from 0.12 ± 0.01 mg/g to 0.33 ± 0.02 mg/g, respectively (Figure 4). The highest content of corosolic acid (1.03 ± 0.04 mg/g) in apple peel samples was found in fruit grown on the semi-dwarf rootstock G.935, which did not differ statistically significantly from the levels found in peel samples of apples grown on dwarf rootstocks EM_04 or 62-396-B10^®^. The highest amount of corosolic acid (0.33 ± 0.02 mg/g) in apple flesh samples was found in fruit grown on the semi-dwarf rootstock EM_01 and did not differ statistically significantly from the amounts found in fruit grown on dwarf rootstocks EM_02 or 62-396-B10^®^. In a study which analyzed the dependence of the growth and quality of apple fruit on the position of the fruit in the fruit tree crown, it was found that the content of corosolic acid varied from 1.25 mg/g to 1.44 mg/g in apple peel samples of the ‘Ligol’ cultivar grown on the rootstock P 60 [31].

**Figure 5:**
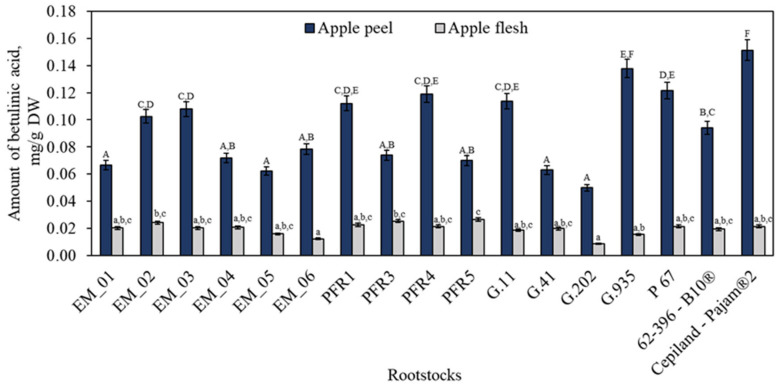
Influence of rootstocks on the quantitative composition of betulinic acid. The means followed by different uppercase and lowercase letters are significantly different at *p* < 0.05.

Betulinic acid content in apple peel and flesh samples was the lowest of all the identified and quantified triterpene compounds. The betulinic acid content in apple peel samples varied from 0.05 ± 0.003 mg/g to 0.15 ± 0.008 mg/g (Figure 5). The statistically significant highest content of betulinic acid (0.15 ± 0.008 mg/g) in apple peel samples was found in fruit grown on the dwarf rootstock Cepiland-Pajam^®^2. The highest amounts of betulinic acid (0.03 ± 0.001 mg/g) in apple flesh samples were found in fruit grown on rootstocks PFR3 and PFR5 (Figure 5). The betulinic acid content ranged from 0.09 mg/g to 0.14 mg/g in apple peel samples of the ‘Ligol’ cultivar grown on the rootstock P 60, reported by Kviklys et al. [31]. In our study, the amount of betulinic acid found in apple flesh samples of the fruit of the ‘Galaval’ cultivar grown on rootstocks corresponded to the content (0.003 mg/g) found in apple flesh samples of the ‘Brookfield Gala’ cultivar [43]. A study revealed a drastic variation among apple clones and concluded that the ‘Maxi Gala’ cultivar had the highest amount of betulinic acid [45].

**Figure 6:**
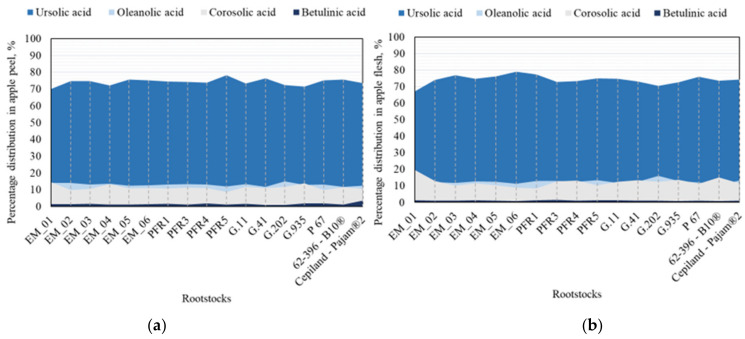
Percentage distribution of individual triterpenes: (**a**) Percentage distribution of triterpenes in apple peel samples; (**b**) percentage distribution of triterpenes in apple flesh samples.

The amount of individual triterpene compounds in apple peel and flesh samples varied depending on the rootstock. In the apple peel samples, ursolic acid accounted for 70.04% to 78.33% of the total triterpene compounds (Figure 6a). Apple flesh samples showed a similar trend in the distribution of ursolic acid (from 67.10% to 79.11%) (Figure 6b). The percentage distribution of oleanolic acid in apple peel and flesh samples was significantly lower. We found that the amount of oleanolic acid ranged from 11.76% to 15.01% of total triterpene compounds in apple peel samples and from 10.52% to 16.11% in apple flesh samples (Figure 6). Depending on the rootstock, the percentage distribution of corosolic acid in apple peel and flesh samples was in some cases higher than that of oleanolic acid. Our study showed that the percentage distribution of corosolic acid in apple flesh samples of fruit grown on the rootstock EM_01 was 19.70% of the total amount of triterpene compounds (Figure 6b). In the apple samples, betulinic acid content accounted for the smallest percentage of the distribution. The study showed that depending on the rootstock, the content of betulinic acid in the apple peel samples could range from 0.98% to 3.43% of the total amount of triterpene compounds, and in the apple flesh samples, from 0.84% to 1.48% (Figure 6). 

The cuticular wax of apple peel represents an important food source of biological active compounds, with ursolic and oleanolic acids being the major components [7]. Since the identification of ursolic acid in apple peels, a series of studies has shown that such a component accounts for up to 32.00% of the total amount of lipophilic compounds in the matrix [37]. In apple flesh, ursolic and oleanolic acids accounted for 79.00–95.00% of the total triterpene content [46].

### 2.2. Cluster Analysis

**Figure 7:**
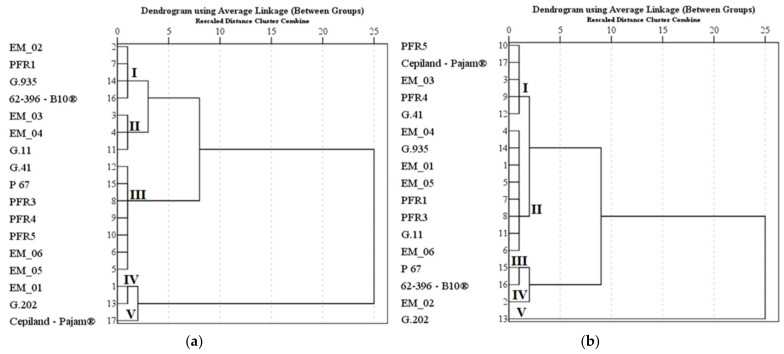
Influence of rootstocks on the quantitative composition of triterpene compounds: (**a**) the dendrogram illustrates variation in the quantitative composition of triterpene compounds in apple peel samples; (**b**) the dendrogram illustrates variation in the quantitative composition of triterpene compounds in apple flesh samples.

We systematized research data on the influence of apple rootstocks on the qualitative and quantitative composition of the total amount of triterpene compounds. In apple peel samples, the total amount of triterpene compounds was divided into five clusters depending on the rootstock (Figure 7a). Cluster I, which had the highest total triterpene levels in apple peel samples, included dwarf rootstocks EM_02 and 62-396-B10^®^ and semi-dwarf rootstocks PFR1 and G.935. Dwarf rootstocks EM_03 and EM_04 developed in the United Kingdom and dwarf rootstock G.11 developed in the United States were included in Cluster II, which had lower total levels of triterpene compounds in apple peel samples. Cluster III, which had average total triterpene levels in apple peel samples, included almost all dwarf rootstocks developed in New Zealand (PFR3, PFR4, and PRF5), dwarf rootstocks EM_05 and EM_06 developed in the United Kingdom, the dwarf rootstock G.41 developed in the United States, and the dwarf rootstock P 67 developed in Poland. Semi-dwarf rootstocks EM_01 and G.202 were assigned to Cluster IV, which had below-average total triterpene levels in apple peel samples. The rootstock Cepiland-Pajam^®^2 developed in France was assigned to cluster V, which had the lowest total triterpene content in apple peel samples (Figure 7a).

In apple flesh samples, the total amount of triterpene compounds was divided into five clusters depending on the rootstock (Figure 7b). Dwarf rootstocks EM_03, Cepiland-Pajam^®^2, PFR4, PFR 5, and G.41 were included in Cluster I, which had average total amounts of triterpene compounds in apple flesh samples. Cluster II, which had below-average total levels of triterpene compounds in apple flesh samples, included almost all the rootstocks developed in the United Kingdom (EM_01, EM_04, and EM_05), the remaining rootstocks developed in New Zealand (PF1 and PFR3), and rootstocks G.11 and G.935 developed in the United States. Dwarf rootstocks P 67 and 62-396-B10^®^ were included in Cluster III, where total levels of triterpene compounds in apple flesh samples were found to be lower than the maximum. The dwarf rootstock EM_02 was assigned to cluster IV, which had the highest total triterpene content in apple flesh samples. The semi-dwarf rootstock G.202 was assigned to cluster V, which had the lowest total triterpene content in apple flesh samples (Figure 7b).

The evaluation of the influence of rootstocks of different growth groups and origin on the qualitative and quantitative composition of triterpene compounds in apple peel and flesh samples of the ‘Galaval’ cultivar showed that the origin of the rootstocks did not have any significant effect on the accumulation of triterpene compounds. We found that the growth group of the rootstocks also did not influence the qualitative and quantitative composition of triterpene compounds. The highest total amount of triterpenes and the highest content of ursolic acid, oleanolic acid, and betulinic acid were found in apple samples of fruit grown on dwarf rootstocks. Dwarfing apple rootstocks are gaining popularity in apple-producing areas across the world because they reduce the vegetative growth of scion cultivars, increase yield, and reduce labor costs for production practices such as spraying, pruning, and harvesting [23]. With the adoption of dwarfing and precocious rootstocks over the past 60 years, apple orchard systems have transitioned from traditional production systems established with large trees in wide spacing arrangements to high-density orchards with smaller closely spaced trees [26]. The studies about the physiological and biochemical mechanisms of dwarfing apple rootstocks have mainly focused on water and mineral transport, hormone levels, anatomical structures, and photosynthetic characteristics [34]. Therefore, our study about the variation in the content of bioactive triterpene compounds in apple peel and flesh samples on rootstocks is important from a practical point of view. To determine the variation in the quantitative composition of triterpene compounds between peel and flesh samples in apples grown on 17 different rootstocks, coefficients of variation were calculated, showing the amplitude of variation for each compound. Depending on the rootstock, the total content of triterpene compounds in apple flesh of the ‘Galaval’ cultivar could vary by 2.35 times and in the peel by up to 75.15%. The genotype of the rootstock resulted in the highest variation in corosolic acid levels in apple flesh (by as much as 2.81 times). On the contrary, in apple peel, the greatest variation was recorded for betulinic acid (by 3.02 times). The most stable individual triterpene compound was ursolic acid, which varied by only 1.80 times depending on the rootstock. The results of this study demonstrated that the rootstocks altered the dynamics of biochemical characteristics. From the health point of view, the concentration of triterpene compounds is considered as an important constituent of apple quality. Thus, the rootstock should also be taken into account as an important factor influencing the concentration of triterpene compounds. A proper matching of the cultivar and the rootstock can program the fruit tree to produce larger amounts of higher quality and high-nutrition-value fruit.

Apple extracts are multicomponent matrices of complex structures, the composition of biologically active compounds of which varies depending on endogenous and exogenous factors. Some evidence is accumulating about phytochemical compounds characterized in vitro and in vivo for their effects on human health. One type of such compounds, the triterpene compounds such as ursolic acid, oleanolic acid, corosolic acid, and betulinic acid, has been suggested to have a broad range of effects on health characteristics [38]. Previous investigations have shown that apples are considered an important source of polyphenols, which are responsible for their well-known antioxidant properties [6,44]. Some authors have stated that triterpenes demonstrate antioxidant activity [47,48]. 

When the natural protection system of an organism including enzymatic, non-enzymatic, or dietary origins is related to the exaggerated generation of reactive oxygen and nitrogen species, macromolecules can suffer oxidative damage, inducing tissue damage [49]. Intake of foods, especially fruits, with a high amount of antioxidant agents is an effective strategy to contend with such tissue damage and objectionable transformations and can prevent the development of chronic diseases. Due to the novelty and for a better understanding of potential new sources of bioactive compounds of apple peel and flesh on rootstocks, it is important in this context to study the contents of triterpene compounds and their antioxidant capacity. Some triterpene compounds function as specific chemical agents against competitive plants, pathogens, or herbivores. Previous studies have described the antioxidant activity of triterpene compounds [8,47]. As it was mentioned, ursolic acid shows a wide range of biological properties, including antioxidant activities. Therefore, it can be a good radical scavenger, a chain-breaking antioxidant, or a chelator of radical-generating metals [48]. Additionally, ursolic acid was able to function as an antioxidant by regulating antioxidant enzymes and protecting key proteins against oxidative stress damage [50]. For instance, several studies have reported on the antioxidant activities of oleanolic acid, but only recently it has been shown not only that oleanolic acid is a free radical scavenger but also that the main antioxidant activity of the molecule is due to the Nrf2-mediated increased expression of antioxidant enzymes such as catalase and thioredoxin peroxidase and enhanced biosynthesis of the antioxidant glutathione [51]. The crude extracts and their fractions of four cultivars (‘Gala’, ‘Golden’, ‘Granny Smith’, and ‘Pink Lady’) enriched in the compounds of interest showed attractive antioxidant activity confirming that apple pomace could be a valuable source of bioactive molecules including not only phenolic compounds but also triterpene compounds [52]. Compared with apple flesh, apple peel shows more potent antioxidant activity and antiproliferative activity, and this can be linked to triterpenes because they are found mostly in apple peel. Apple peel directly exposed to (a)biotic stress resulted in a faster synthesis of bioactive compounds and stronger antioxidant activity of apple peel extracts compared to apple flesh samples [18]. Rootstocks improve tolerance to various environment stresses by producing different types of antioxidants that scavenge or detoxify reactive oxygen species. These antioxidants are health-promoting phytochemicals, and their accumulation increases the health value and quality of fruits [53,54].

Some evidence is accumulating about phytochemical compounds characterized in vitro and in vivo for their effects on human health. Triterpenic acids such as ursolic acid, oleanolic acid, corosolic acid, and betulinic acid have not only antioxidant properties but also a broad range of effects on health characteristics [38]. The data of other studies have shown that ursolic acid protects hepatocytes and mucous membranes and has a strong anti-inflammatory, antibacterial, antiviral, and glycemia-lowering effect [55,56,57]. One of the important pharmacological properties attributed to oleanolic acid is its hepatoprotective effect. It has been shown that oleanolic acid is not only effective in protecting the liver from acute chemically induced liver injury but also protects the liver from fibrosis and cirrhosis caused by chronic liver diseases [58]. A previous study described that corosolic acid increased GLUT4 translocation in mouse muscle and improved glucose metabolism by reducing insulin resistance [59]. A variety of biological activities have been ascribed to betulinic acid including anti-inflammatory effects, selective cytotoxicity against several melanoma-derived cell lines, and in vitro anti-malarial effects [60].

Triterpene compounds in apples grown on rootstocks are interesting agents from the pharmaceutical and nutraceutical points of view, as well as due to their importance in plant biochemistry. A properly selected combination of rootstock and cultivar can affect the fruit, enriching it with biologically active compounds and antioxidant activity.

## 3. Materials and Methods

### 3.1. Plant Material

In this study, we evaluated the apple cultivar ‘Galaval’ grown with 17 rootstocks of different growth groups (Table 1). The orchard was planted at the Institute of Horticulture (Babtai, Lithuania), a branch of the Lithuanian Research Center for Agriculture and Forestry (coordinates: 55°60′N, 23°48′E) in spring 2017 in the frame of a multi-location apple rootstock trial led by the EUFRIN (European Fruit Research Institute Network) Apple & Pear Variety & Rootstock Testing Working Group. The planting material was propagated as 1 year-old trees by the Dalival nursery, France. Planting distances depended on rootstock vigor: 3.5 × 1 for the dwarf rootstocks and 3.5 m × 1.5 m for the semi-dwarf rootstocks. Each rootstock was replicated four times, with three trees per plot. Trees were trained as slender spindles. A sustainable plant protection system was used for orchard management [61]. Apples of the ‘Galaval’ cultivar were harvested at optimal fruit maturity considering suitable external fruit appearance, fruit size, and colors. The study was conducted during 2020–2021.

### 3.2. Chemicals and Solvents

All solvents, reagents, and standards used were of analytical grade. Acetonitrile, acetone, ursolic acid, oleanolic acid, betulinic acid, and corosolic acid were obtained from Sigma-Aldrich GmbH (Bethesda, MD, USA). Purified deionized water used in the tests was prepared with the Milli-Q^®^ (Millipore, Bedford, USA) water purification system. Reagents used in the antioxidant activity assay—6-hydroxy-2,5,7,8-tetramethylchroman-2-carboxylic acid (Trolox), 2,2-diphenyl-1-picrylhydrazyl (DPPH), and sodium acetate—were obtained from Scharlau (Barcelona, Spain), 2,4,6-tri(2-pyridyl)-s-triazine (TPTZ) was obtained from Carl Roth (Karlsruhe, Germany), and iron (III)) chloride hexahydrate (FeCl_3_ × 6H_2_O) from Vaseline-Fabrik Rhenania (Bonn, Germany).

### 3.3. Preparation of Apple Lyophilizate and Apple Extracts

Apple flesh and peel samples and their extracts were prepared as described by Butkeviciute et al. [62].

### 3.4. Estimation of Triterpenes by the HPLC-PDA Method

Qualitative and quantitative high-performance liquid chromatography (HPLC) analyses of triterpenes in the apple flesh and apple peel extracts were conducted. A chromatograph equipped with a PDA detector Waters 2998 (Waters, Milford, CT, USA) was used for high-performance liquid chromatography (HPLC) analysis. Chromatographic separations were carried out by using an ACE (5 μm, C18, 250 × 4.6 mm inner diameter) column. The column was operated at a constant temperature of 25 °C. The volume of the analyzed extract was 10 μL. The flow rate was 1 mL min^−1^. The mobile phase consisted of acetonitrile (solvent A) and water (solvent B). We applied isocratic elution, the eluent ratio being 88% (solvent A) and 12% (solvent B). For the quantitative analysis, the calibration curve was obtained by injecting known concentrations of different standard compounds. All the identified triterpenes were quantified at 205 nm wavelength [63].

### 3.5. Data Analysis

The analysis of the data of the HPLC method was performed using Microsoft Office Excel 121 (Microsoft, Redmond, WA, USA) and SPSS, version 25.0 (SPSS Inc., Chicago, IL, USA). All the results obtained during the HPLC analysis are presented as means of three consecutive test results and standard deviations. The univariate analysis of variance (ANOVA) was applied in order to establish whether the differences between the compared data were statistically significant. The hypothesis about the equality of variances was verified by applying Levine’s test. If the variances of independent variables were found to be equal, Tukey’s multiple comparison test was used. The differences were regarded as statistically significant at *p* <0.05. The comparison of the chemical composition between the apple samples was carried out by applying the hierarchical cluster analysis, using the squared Euclidean distance.

## 4. Conclusions

The rootstock–scion combination had a significant effect on the qualitative and quantitative composition and in vitro antioxidant activity of triterpene compounds in apple flesh and apple peel samples. The results obtained did not depend on the origin or the growth class of the rootstocks. The highest total content of triterpene compounds was found in peel samples of apples grown on the dwarf rootstock 62-396-B10^®^. Depending on the rootstock, apple peel samples accumulated 3.52 to 4.74 times more triterpene compounds than apple flesh samples. Ursolic acid was the predominant triterpene compound in apple peel and flesh samples. The highest content of ursolic acid was also found in fruit grown on the dwarf rootstock 62-396-B10^®^. 

A proper match of a cultivar and a rootstock can program a fruit tree to grow larger amounts of higher quality, antioxidant-rich, and high-nutrition-value fruit. Such combinations can be used when designing modern gardens, and the fruit would be essential for practical and fundamental medicine and in the production of novel supplements, foods, or other products.

## Figures and Tables

**Table 1:** Origin and growth characteristics of apple rootstocks.

No.	Rootstock	Country of Origin	Vigor According to Breeders	Actual Vigor
1.	EM_01	UK	Semi-dwarf	Semi-vigorous
2.	EM_02	UK	Dwarf	Small dwarf
3.	EM_03	UK	Dwarf	Small dwarf
4.	EM_04	UK	Dwarf	Super dwarf
5.	EM_05	UK	Dwarf	Small dwarf
6.	EM_06	UK	Dwarf	Semi-dwarf
7.	PFR1	New Zealand	Semi-dwarf	Semi-dwarf
8.	PFR3	New Zealand	Semi-dwarf	Semi-dwarf
9.	PFR4	New Zealand	Dwarf	Semi-dwarf
10.	PFR5	New Zealand	Dwarf	Dwarf
11.	G.11	USA	Dwarf	Strong-dwarf
12.	G.41	USA	Dwarf	Strong-dwarf
13.	G.202	USA	Semi-dwarf	Semi-dwarf
14.	G.935	USA	Semi-dwarf	Strong-dwarf
15.	P 67	Poland	Dwarf	Dwarf
16.	62-396-B10^®^	Russia	Dwarf	Dwarf
17.	Cepiland-Pajam^®^2	France	Dwarf	Strong-dwarf

## Data Availability

All datasets generated for this study are included in the article.
